# Xanthogranulomatous Pyelonephritis of a Lower Pole Moiety in a Duplicated Collecting System Kidney

**DOI:** 10.1155/2015/506078

**Published:** 2015-09-08

**Authors:** Jean Paul Wuilleumier, Ariel Schulman, Antonio Montgomery, Ervin Teper

**Affiliations:** ^1^General Surgery Residency Program, Maimonides Medical Center, 4802 10th Avenue, Brooklyn, NY 11219, USA; ^2^Urology Residency Program, Maimonides Medical Center, 4802 10th Avenue, Brooklyn, NY 11219, USA; ^3^Urology Department, Maimonides Medical Center, 4802 10th Avenue, Brooklyn, NY 11219, USA

## Abstract

Xanthogranulomatous pyelonephritis (XGP) is a destructive inflammatory process which is frequently caused by recurrent urinary tract infections or chronic obstruction by kidney stones. We present a 56-year-old female with an extensive retroperitoneal urinoma and xanthogranulomatous pyelonephritis of the lower pole moiety in a kidney with a duplicated collecting system due to obstructive nephrolithiasis. After drainage of the urinoma, the patient underwent a definitive lower pole heminephrectomy with preservation of the functional upper pole. We review important clinical features of xanthogranulomatous pyelonephritis and considerations for surgery on a duplicated kidney.

## 1. Introduction

Xanthogranulomatous pyelonephritis (XGP) is a destructive inflammatory process defined by parenchymal infiltration by histiocytes and lipid-filled macrophages [1]. It occurs most frequently in immunocompromised, middle-aged females with chronic or recurrent urinary tract infections and obstruction by kidney stones. The classic triad is an enlarged kidney, nephrolithiasis, and poor renal function [2]. Surgery is the mainstay of therapy. We present a 56-year-old female with xanthogranulomatous pyelonephritis of the lower pole moiety in a kidney with a duplicated collecting system due to obstructive nephrolithiasis successfully managed with a lower pole heminephrectomy.

## 2. Case Report

A 56-year-old African American female with a past medical history of colon cancer of 3 years of status post-low-anterior-resection and chemotherapy presented to the emergency room with 3 months of progressive, debilitating left hip pain, 40-pound weight loss, and weakness. She denied flank pain, hematuria, or bright red blood per rectum. On exam, the patient was afebrile, with a heart rate in the 110s and systolic blood pressure in the 90–100s. She appeared cachectic. On abdominal exam there was a well-healed low midline incision. There was no flank pain but there was left lower quadrant discomfort and limited range of motion of the left leg with associated hip pain. WBC count was 14,900, hemoglobin was 6.5 g/dL, hematocrit was 20.1%, and the platelet count was 462,000. BUN was 19 and creatinine was 1.4. The urinalysis was significant for a pH of 7.0, moderate hemoglobin, and large leukocyte esterase with negative nitrites. There were >50 WBCs and 10–25 RBCs and many bacteria. Urine culture was 20,000 CFU with more than 3 organisms present. Blood culture was positive for vancomycin sensitive* Enterococcus faecalis*.

**Figure 1:**
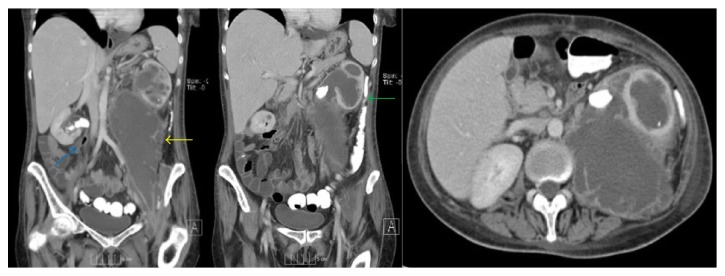
Abdominal CT scan on initial evaluation revealed a large enhancing retroperitoneal fluid collection measuring 11.5 × 22.0 cm (yellow arrow) extending into entire left psoas muscle and hip region. There were bilateral complete duplicated collecting systems with bilateral obstructing large UPJ calculi in both lower pole moieties. The left kidney was remarkable for moderate hydronephrosis of the upper pole moiety and severe cortical thinning of the lower pole. The left kidney was duplicated with moderate upper pole hydronephrosis and moderate-to-severe hydronephrosis with cortical thinning of the lower pole moiety (green arrow). The right kidney had preserved parenchyma with associated emphysematous pyelitis (blue arrow).

CT scan of the abdomen and pelvis with intravenous contrast revealed a 22 cm × 11 cm enhancing left retroperitoneal fluid collection extending along the length of the psoas muscle and tracking into the left hip. The left kidney had a duplicated collecting system with moderate hydronephrosis of the upper pole moiety. The renal pelvis of the lower pole moiety was obstructed with a 3 cm stone with apparent loss of integrity of the renal pelvis with urine extravasation. The lower pole parenchyma had calyceal ballooning and significant cortical thinning. The right kidney also had a duplicated collecting system with a preserved upper pole parenchyma and no hydronephrosis. The lower pole renal pelvis was obstructed with UPJ calculi and a focus of air consistent with emphysematous pyelitis. There was dilation of the proximal renal pelvis without parenchymal atrophy (Figure 1). These abnormal CT findings were highly of chronic pyelonephritis/XGP secondary to chronic obstruction versus less probable malignant transformation.

**Figure 2:**
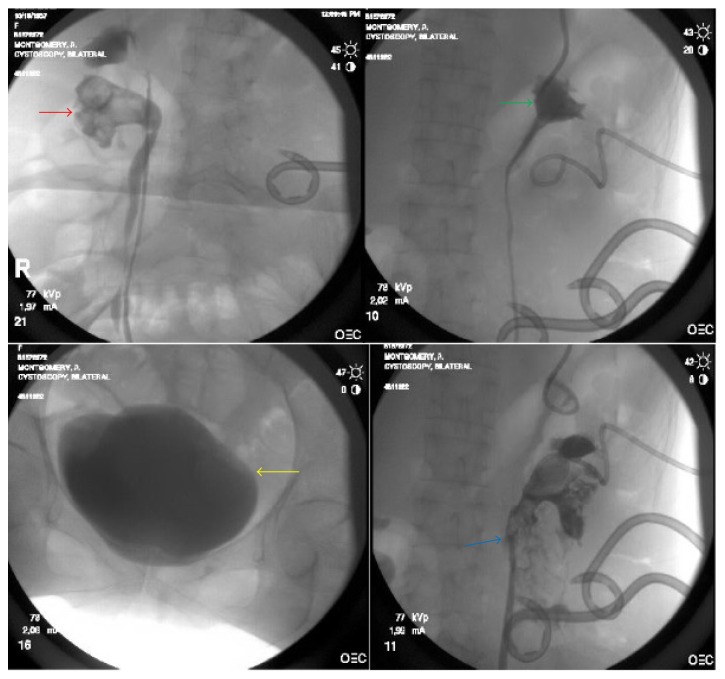
Preoperative cystoscopy with bilateral retrograde pyelograms shows a bladder without congenital anomalies (yellow arrow), a classic “drooping lily” sign of the right lower pole moiety (red arrow), an independent left upper pole moiety without abnormalities (green arrow), and a left lower pole containing large UPJ calculi with perforation of its renal pelvis and leakage of contrast material into retroperitoneal space (blue arrow).

**Figure 3:**
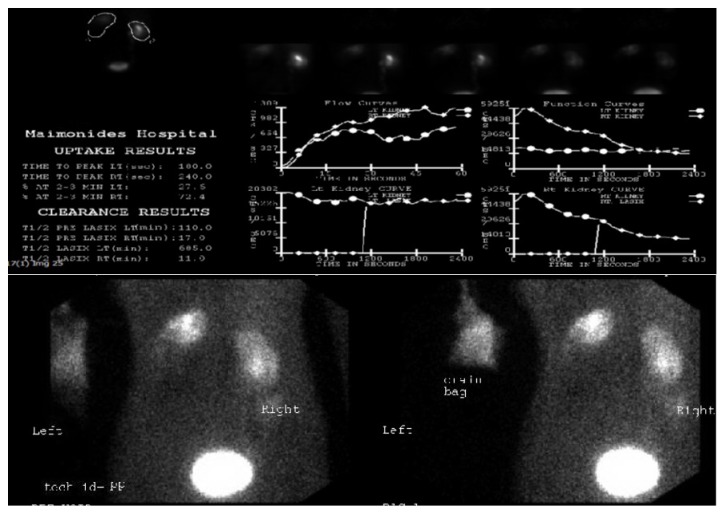
Nuclear Mag-3 renal scan demonstrated split renal function of left kidney 28% and right kidney of 72% with severe renal dysfunction of left lower pole moiety and a normally functional right kidney.

The patient was admitted to the hospital, resuscitated, and covered with broad-spectrum antibiotics. Two large bore retroperitoneal drains were placed percutaneously with drainage of purulent fluid. Abscess culture was significant for* Proteus* spp. and* E. faecalis*. Percutaneous nephrostomy tubes were placed in both the upper and lower pole moieties. Following defervescence, the patient was taken to the fluoroscopy suite for further evaluation. Retrograde pyelography confirmed complete bilateral ureteral duplication with no vesicoureteral reflux. There was contrast extravasation from the left lower pole moiety confirming urine leak from the renal pelvis. The lower pole of the right renal pelvis had significant stone burden but preserved integrity (Figure 2). On Mag-3 renal scan with furosemide washout, the left kidney contributed with 28% of overall renal function, with approximately 90% of that from the upper pole moiety. There was minimal contrast uptake in the lower pole (Figure 3).

The patient was taken to the operating room for an open left retroperitoneal washout and open lower pole heminephrectomy. A stent was placed in the left upper pole ureter to assist with intraoperative identification and preservation. Via a left flank incision, the left retroperitoneum was explored and significant fibrosis and inflammatory rind was encountered around the lower pole of the kidney. The lower pole was completely mobilized and a common sheath with both ureters was identified. The sheath was opened and the stented upper pole ureter was identified and excluded. The lower pole ureter was traced cranially to the renal pelvis and a 2 cm necrotic defect was noted in the collecting system. The pelvis was opened and several large stones were removed. The lower pole parenchyma was markedly thinned and amputated. The exposed cortex was fulgurated with cautery and the defect was reapproximated with liver sutures and hemostatic agents. Significant necrotic rind was washed out of the retroperitoneal space along the psoas muscle and a large bore drain was left in place. The patient tolerated the procedure without complication.

The gross pathologic specimen consisted of several fragments of irregular, pink-brown kidney with orange-white discoloration with white fibrous surfaces and multiple pale yellow lesions with scalloped borders. The uninvolved parenchyma was pink-tan and smooth. Additional fragments of dusky green-yellow necrotic tissue and calculi were also submitted. Microscopic examination of the kidney fragments revealed focal xanthomatous changes and obstructive uropathy. The necrotic fragments showed acute inflammatory exudate with fibrin residues.

**Figure 4:**
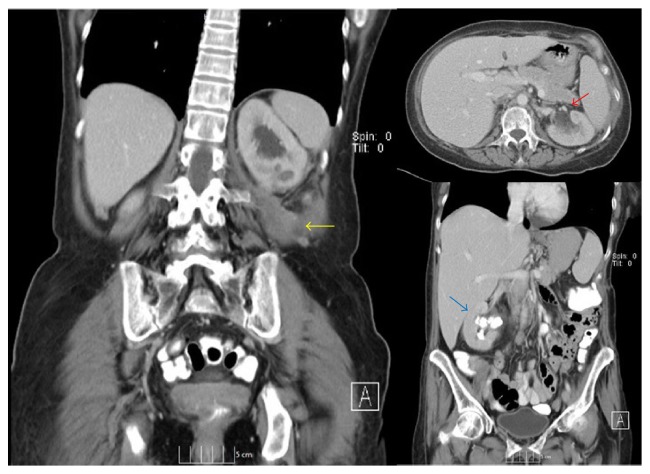
Postoperative CT scan performed 2 months after surgery showed a small residual retroperitoneal collection measuring 2.1 × 1.0 cm (Yellow arrow) and interval resolution of left upper pole hydronephrosis (red arrow) and a right kidney with a nonobstructive large UPJ calculus (blue arrow).

The patient was discharged from the hospital several days after surgery with significant improvement in left hip pain. Interval CT scan 2 months after surgery revealed almost complete resolution of the retroperitoneal abscess and preservation of the left upper pole moiety (Figure 4).

## 3. Discussion

Xanthogranulomatous pyelonephritis (XGP) is a destructive inflammatory process defined by parenchymal infiltration by histiocytes and lipid-filled macrophages [1]. It occurs most frequently in immunocompromised, middle-aged females with chronic or recurrent urinary tract infections and obstruction by kidney stones. The classic triad is an enlarged kidney, nephrolithiasis, and poor renal function [2].* Escherichia coli* and* Proteus* species are the most commonly isolated infectious agents [2]. On CT scan, a “bear paw” sign may be appreciated with overlapping dilated calyces with a thinned parenchyma [3].

Surgery is the mainstay of therapy but can be notoriously difficult with the extension of the inflammatory process to nearby structures being common. A laparoscopic approach is safe in select cases, although with longer operative times and a higher anticipated rate of conversion to open surgery [4, 5]. Most cases are managed with nephrectomy, but nephron-sparing surgery has been reported in select patients [6]. If an attempt at nephron-sparing surgery is planned, preoperative drainage of infected fluid and the urinary tract seems prudent. We performed surgery after demonstrating a period without fevers and with a normal white blood cell count. We also found that preoperative placement of the upper pole stent was particularly helpful to identify and preserve the upper pole ureter and advocate this maneuver in the surgical management of duplicated kidneys.

In consistency with previous case reports, our case of XGP was diagnosed by histological analysis after surgical resection. We would like to emphasize that one of the many differential diagnoses in this patient could have easily been malignancy due to her symptoms of weakness and progressive weight loss. This patient's CT findings could be interpreted as malignant transformation. Pérez et al. reported a case of XGP in the obstructed upper pole moiety of an infant, but this is the first report in the literature of XGP isolated to the lower pole moiety of a duplicated renal unit [7]. While most duplex kidneys are asymptomatic, the classic pathologic finding is obstruction of the upper pole moiety from an inferior and medial ectopic ureter and vesicoureteral reflux of the lower pole moiety from a superior and laterally inserting ureter [8]. Here we noted both ureters along the trigone in the bladder, the absence of vesicoureteral reflux on cystogram, and no evidence of long-standing obstruction of either upper pole unit. A noteworthy feature of this clinical case was the unusual location of this patient's XGP as previously reported cases usually presented in the upper pole of duplicated kidneys. The chronic inflammation due to prolonged obstruction most likely led to this degeneration of the renal parenchyma. An additional point of interest was the presence of similar obstructive nephrolithiasis and emphysematous pyelitis of the lower pole moiety of the right kidney. We are planning percutaneous nephrolithotomy for management of the right lower pole stones.

We were particularly pleased with the follow-up CT 2 months after surgery with almost complete resolution of the retroperitoneal abscess and preservation of the functional upper pole of the kidney. This confirmed the safety of upper pole conservation in this unique clinical scenario.
